# Applying Lean in Process Innovation in Healthcare: The Case of Hip Fracture

**DOI:** 10.3390/ijerph17155273

**Published:** 2020-07-22

**Authors:** Manuel Francisco Morales-Contreras, Pedro Chana-Valero, Manuel F. Suárez-Barraza, Andrés Saldaña Díaz, Elena García García

**Affiliations:** 1 Department of Management, ICADE, Universidad Pontificia Comillas, ICADE, 28015 Madrid, Spain; 2 Fundación San Juan de Dios, Centro de CC de la Salud San Rafael, Universidad Nebrija, 28036 Madrid, Spain; pchana@nebrija.es (P.C.-V.); egarciga@nebrija.es (E.G.G.); 3 International Business Department, School of Business and Economy, Universidad de las Américas Puebla (UDLAP), Puebla 72810, Mexico; manuel.suarez@udlap.mx; 4 Hospital San Juan de Dios León, 24010 León, Spain; ansaldiaz@gmail.com

**Keywords:** lean, process innovation, healthcare, quality improvement, hip fracture integrated care pathway

## Abstract

Academic literature and practitioners acknowledge that there is a need to improve efficiency and service quality in the healthcare industry. In Spain, osteoporotic fractures represent a great cost in socio-economic and morbi-mortality terms, hip fracture being the surgical pathology with the second highest consumption of resources. The research questions that govern this study concern the use of Lean principles to identify waste, and an evaluation of the application of an innovative approach in the hip fracture surgery process. A research design based on a case study and action research was developed. Findings relate to (i) the identification of the main types of waste or *muda* (being the most frequent delay, transportation, over-processing and defects); (ii) the analysis of existing processes based on a Lean approach (identifying opportunities for improvement as a reduction of the number of steps and participants, improving communication, automation, standardization, etc.); and (iii) the application of an innovative process based on the Lean approach and action research in the healthcare industry. This research provides insights for academia, practitioners, management, and society: waste identification and process redesign helps to continue the improvement of operations, increase efficiency, reduce costs, and enhance services, providing benefits to patients, families, hospital employees, and the healthcare system.

## 1. Introduction

Among the high-risk industries, healthcare is the most poorly managed of all and is very late in coming to recognize the importance of the system factors that underlie adverse events [1], making a healthcare system analysis and support for patients and staff an absolute priority [2]. Overtreatment, undertreatment, overdiagnosis, underdiagnosis, uncontrolled costs and budgets, and medical treatment errors have been reported in health systems across the developed world [3]. Medical error can be defined as a deviation from the process of care that may or may not cause harm to the patient [4]. In the USA, medical errors are the third cause of death after cancer and heart disease. In Spain, it is estimated that medical errors were involved in 25.9% of court verdicts, 98.5% of them resulted in compensation awards [5], leading to an increase in healthcare costs. But not only medical errors impact patient experience and lack of efficiency in healthcare; other examples are: delays, long waiting times, waiting lists for service delivery, lack or excess of capacity, lack or excess of inventory, patients going to the hospital several times until a service is provided, unsatisfied patients, etc. The management of a healthcare organization needs to be able to make decisions on the value delivered to patients and relatives, so patient value must be the key to making responsible decisions as a health system. Porter defines patient value as the patient-relevant medical outcome divided by cost [3].

Funding and efficiency in the health sector becomes a priority issue in the context of an ageing population [6]. To address the crisis, the NHS (National Health Service, UK) has turned to the use of different “quality improvement” methodologies, often discovered outside the health sector, Lean management systems being one of them [7].

The term Lean applied to production or management systems was used for the first time by Womack et al. [8] It was derived from the Toyota Production System, which was oriented to the continuous improvement of quality, productivity, and efficiency, as well as to the reduction of costs and delivery times within the Japanese automotive industry [8,9,10,11,12]. Lean is doing more with less and refers to a methodology that pursues the identification and elimination of waste (*Muda* in Japanese) [8]. Waste is defined as any activity that does not add any value from the customer perspective, thus reducing the efficiency of a process and increasing its costs [9,11]. Literature states that seven types of waste could be found in any process: defects, movements, process, inventory, overproduction, transportation, and time [8,9,11,12].

Although Lean management systems were originated within the manufacturing sector, there is a growing interest for its implementation in service companies [13]. There are many opportunities of finding waste in the service sector as the processes tend to be slow, and tend to include high values of work in process (or customers waiting), which has an impact on the costs, service quality, and customer satisfaction [14].

Lean practices, with the aim of improving medical care processes, can help to achieve more reliable healthcare systems by addressing the three steps for reducing system errors in healthcare, proposed by Makary et al., in 2016 [15]: making errors more visible (facilitate a culture of speaking up), responding to error (support clinician needs), and making errors less frequent (foster culture of safety). Lean has been implemented in different areas in healthcare as intensive care units, X-Ray, cardiology surgery, oncology, mental health units, and clinical laboratories [7,16,17,18,19,20]. A national survey in the United States found that 70% of hospitals used Lean healthcare or related methodologies to deal with both quality and cost issues [21]. Gonzalez-Aleu et al. in 2018 analysed the critical success factors when implementing continuous improvement projects in hospitals [22]. But there is not enough evidence to address its sustainability in broad healthcare adoption as well as its impact in quality healthcare [7] and achieve both effectiveness and efficiency improvements [18]. Thus, it remains a “challenge for academics and practitioners to evaluate Lean healthcare under a more critical perspective” [17].

Hip fracture is one of the leading pathologies in terms of resource consumption [23], with a progressive increase in the estimate of new cases per year [24] and a high-risk patient profile. Applying Lean to this scenario, with the aim of identifying barriers, sensitized with the patient’s needs and mapping the experience of the different actors involved, can improve quality of care and increase the efficiency of processes, helping managers and staff address more complex issues and deepen our understanding of how Lean works in healthcare [25].

The main research questions that govern the study are:

RQ1. → What types of waste or *muda* could be identified around a hip fracture surgery process?
RQ2. → How a process innovation methodology using Lean techniques is applied in healthcare, in particular in a hip fracture surgery processes?

With the aim of answering these research questions, we decided to conduct a methodology based on a case study and action research, by involving the research team in the process evaluation and redesign. The article is structured as follows: (i) an introduction; (ii) a literature review and theoretical framework; (iii) a description of the research methodology; (iv) the case study results; (v) discussion; and (vi) conclusion.

## 2. Theoretical Framework

Hip fractures are potentially catastrophic (deadly) events with adverse outcomes including alteration in function, institutionalization, and death [26]. Mortality rates have increased in ageing patients who live alone and who have poor pre-fracture mental status and mobility [26,27,28], other associated factors being: white race, osteoporosis, previous hip fracture, level of physical functioning, medication use, and hormonal and dietary factors [29]. Osteoporosis fractures are the fourth leading cause of chronic disease morbidity. Over 2 million women and 750,000 men are estimated to suffer osteoporosis at the level of the femoral neck, with an expected prevalence of 24.2% in women between 70 and 80 years old. The cost to the Spanish healthcare system related to osteoporosis fractures amounted to 4.2 billion euros in 2017 and is expected to increase to 5.5 billion in 2030 [30].

More than 500,000 acute hip fractures will occur annually by 2040; each year at least 300,000 old people are hospitalized by hip fractures [24]. The number of geriatric hip fractures is expected to rise annually all over the world related to the elderly population ages, which will significantly increase care costs for healthcare systems, patients, relatives, and societies. The incidence of this complication for the elderly in the United States is expected to grow to half a million by 2040, with a worldwide incidence of geriatric hip fractures reaching up to 4.5 million by 2050 [31,32,33]. In Spain, hip fractures are the second highest obstetric and surgical condition in resource consumption after colorectal and anal cancer regarding the average costs of the Diagnosis Related Groups (DRG) [23], with an estimation of 263,351 new hip fractures in women and 84,213 in men between 2010 and 2020, with an existing tendency to increase in older age groups (>80 years) [34]. In the 2018 National Registry of Hip Fractures (RNFC) report, the overall profile, of the 11,431 patients included from the 72 participating hospitals, described a profile of patients with an average age greater than 87 years, usually living at home, with 76% being women [35].

Looking for efficiency in the process at hand, with a mortality rate within the first year at 20–33% (that may persist beyond 5 years) is a priority, as is relying on value-based healthcare models [31,32]. In the first three months after surgery, older adults have an eight times higher risks of dying of a hip fracture compared with people who have not suffered from it, continuing the risk of death still in the first ten years [36]. Related to functional outcomes, which directly impact the patient´s social dimension, it is important to describe functional outcomes such as the inability to walk independently (40% of hip fractures patients), the situation of vulnerability, needing assistance to perform daily tasks (60%) and patients who become totally dependent requiring a nursing home one year after fracture.

Social impact is relevant, according to a study published in the British Medical Journal, which detected 80% of women over 75 years who would prefer to die rather than experience the loss of independence and living in a nursing home after hip fracture discharge [37].

According to Bradeanu et al., health and social care for hip fractures in the elderly in one year amounts to two billion euros in the UK, where the annual incidence was 79,243 euros in 2019, expecting to increase to 104,000 cases by 2025. Dementia incidence will reach 75.6 million in 2030 and 135.46 in 2050 in the whole population, but doubles every five years among people aged 65 to 90. They also highlight the social impact that both pathologies together have on the caregivers, including depression, a higher risk of developing anxiety, and more hours per day spent helping patients. Furthermore, hip fractures are associated with the development of disability, depression, and cardiovascular diseases in the elderly, with additional cost for society [36].

Therefore, with an increasing incidence and associated poor clinical outcomes, it is relevant to evaluate the impact of hip fractures in the elderly not only on the healthcare system but also in terms of the social impact that is generated by patients as well as by their relatives, the society and the environment [32]. Living in changing times regarding healthcare, political agendas, budgetary limitations, and new leaderships contributes to demanding from professionals the provision of greater value with fewer resources, promoting a bigger impact on patients and other stakeholders [38].

Nowadays, patients are increasingly demanding immediate, high-quality, and individualized care to their specific needs. To meet these demands, strategies are needed to combine sustainable quality improvement with increased efficiency [39]. Improving and ensuring effectiveness of clinical procedures is necessary, but so is improving healthcare performance using appropriate indicators: what works, for whom, and in which circumstances? This must be addressed to not leave anyone behind [40,41].

In a progressive ageing population scenario, indicators that measure the social value of interventions are becoming key indicators to be analyzed in a process of quality continuous improvement. It is important to detect key performance indicators during the process analysis so that, once improvements have been implemented, they can be measured; because collecting data is not enough, they must be used to improve healthcare [40].

Despite being the focus on which to work, currently the most important indicators to measure the performance of the health care process are the length of hospital stay (LOS), i.e., the number of days comprised between the date of a patient admission and the discharge date [42], as well as the intra-hospital, 30-day, and year-round survival. Exploring Lean healthcare on patient flow, the main outcomes categorized related to the utilization of services and access to services [21,43] are the length of stay, mean waiting time to see a doctor, mean waiting time to get treatment, median time for daily treatment, median waiting time to see a professional (nurse preparation), and the median process time of being discharged. Therefore, LOS and shorter waiting times after Lean healthcare intervention were the most common measures related to process outcomes. Related to the hip fracture care pathway, usual measurements are time to surgery, duration of surgery, detection of complications, hospital cost, allogenic transfusion rate, thirty-day readmission, and in-hospital mortality [44,45].

The provision of quality health services depends on an adequate and efficient execution of each of the processes in which not only clinical activities exist, but also in which a series of non-clinical support activities are executed by different types of resources, which varies from one organization to another. These processes are highly complex and dynamic, and it is becoming increasingly common to design them ad hoc with a multi-disciplinary character but, seeking a balance in the improvement of processes between the generation of impact on patient´s quality of life on the one hand and the need to reduce costs, reduce waiting times, and to improve the productivity of each resource on the other, is not an easy task [46].

In 2012, the European Commission proposed a new cooperation framework for innovation in the field of active and healthy ageing, adopting the “Quadruple Helix” innovation model, which aims to generate shared value involving and benefiting civil society, private companies, academia, and the public sector, thus creating a powerful innovation ecosystem [47]. Designing strategies to implement effective solutions according to this model requires the use of specific tools that can trigger improvements in the management and handling of risks, including psychosocial risk management and, subsequently, better well-being [48]. A useful method is the “design thinking” or user-centred design, proposed by the Stanford University Institute of Design, which establishes five unavoidable phases in the process of designing a digital solution, so that it is finally adopted by the target audience: empathizing, defining, devising, prototyping, and evaluating [47].

A more sceptical and scientifically rigorous approach to the development, evaluation, and dissemination of quality improvement methodologies is required, combined with the demand of more robust evidence for the methods and approaches that they use, in those areas of knowledge where a mix of theoretical, empirical, and experimental evidence is used to enable guidance and planning for their application [49].

Strategies focused on the improvement of care quality, waiting times, resource consumption, etc., with the aim of assuring sustainable high-quality care, are needed. For instance, innovative methods such as design thinking combined with Lean. Lean has proved to be a method that has become, in the past decade, one of the most commonly used as a quality improvement approach in healthcare settings to improve delivery of care [25,50], guaranteeing the improvement of the effectiveness and efficiency of health care delivery, and providing an impetus for establishing the best practice within an organization [42].

The Lean methodology approach was developed in the car manufacturing industry, and was later adopted in the healthcare field with the aim of improving quality of care and the efficiency of processes [25,39,51]. The first authors to carry out research on Lean in health were Young et al. in 2004, and Spear in 2005 [52,53]. Both argue that carrying out Kaizen and Lean Thinking efforts in health systems can help eliminate errors, delays, inadequate processes, duplications, and all kinds of MUDA in the activities of health services. Another pioneering research work on the subject was that of Kollberg and Dahlgaard et al., in 2007, who emphasize in their article that continuous improvement techniques help to significantly improve the performance of processes and services in health systems (specifically in Sweden in this case) [54]. Drotz and Poksinska also confirm the benefits of implementing Lean and Kaizen in health organizations because it generates positive effects on the positions and roles of medical personnel, moving from a bureaucratic style to an approach of agile processes and teamwork [20]. On the other hand, authors such as Bortolotti et al. have found 14 specific factors that increase the ability of employees to solve problems when using Kaizen in health systems [55]. The clarity of goals, the degree of the difficulty of objectives, the autonomy of the work teams, and the support of the top managers are critical to the success of the application of Kaizen according to these researchers.

On the other hand, Ortíz-Barrios and Alfaro-Saiz (2020) carried out a literature review of the application of process improvement in emergency processes in health hospitals [56]. The selected papers were categorized considering the leading ED problems and publication year. Two hundred and three (203) papers distributed in 120 journals were found to meet the inclusion criteria. In Latin America, Brazilian authors such as Coehlo et al. (2015) present a case study of process improvement, in which the performance improvement of the total workspace was 75% and the reduction in waiting for patient care went from 2 h to 30 min [57]. Coelho et al. (2015) also point out that Lean and Kaizen’s efforts can eliminate at least three hours a day of overtime in hospitals in Brazil. Curatolo et al., in 2014, also performed a literature review indicating that a Lean approach with a high-methodological maturity level that includes the 11 characteristic activities of process innovation or Business Process Improvement has never been reported [58]. Considering this, the paper suggests a meta model for a high-methodological maturity-level Lean method based on the characteristic activities of Business Process Improvement. Finally, Meyer et al. present the successful application of Lean Six Sigma, a set of quality improvement (QI) tools, to streamline their processes and uncover the root causes of program inefficiencies. All this for a hospital that performs treatments for cancer patients by Tobacco [59].

Godley et al. affirmed that quality improvement studies improving timeliness in healthcare are essential for reducing delays in care and for improving quality [60]. In 2018, Woodnutt et al. carried out a systematic literature review on the Lean sustainable method in NHS hospitals, finding that waiting times were the most common area in which Lean practices could have an effect [7]. In the management of patients undergoing hip replacement surgery, recent studies applying Lean methodology in combination with other strategies improved quality and at the same time reduced costs, resource consumption, and waiting times [42,44]. Moreover, interventions aimed at improving pre-fracture function and post-fracture social support could increase health perception following hip fracture [61] and there is evidence that psychological and social factors, particularly social support, influence recovery and post-fracture quality of life [62].

Therefore, Lean methodology, with the aim of identifying fundamental areas of delay and inefficiency throughout the process, has not been fully implemented to hip fracture care individually or combined with other methodologies [44], such as design thinking. This would enable the mapping of the patient’s experience (journey map) during hospital admission and subsequent discharge from the perspective of what he/she sees, hears, and feels at each phase of the process [63].

Lean methodology is used to increase value in healthcare, but it is seen that the determination of value is variable. It is not easily quantified under evaluation of healthcare-related services, maybe because much of this value is not based on clinical outcomes but on social ones, which are difficult to describe, capture, and translate into a decision-maker´s language, usually financial [38]. There is no standardized way to capture the social value inherent in healthcare programs, but we must work to obtain not only the direct and indirect costs, but also to determine the impacts they are creating.

Lean and Kaizen are focused on improving processes towards an ideal state, with the focus always on adding value to the client (patients), identifying waste (tasks that do not benefit or add value), reducing costs, and improving the work of professionals [7,48]. The origins of business process innovation could be traced back to the seminal works of Harrington [64] and Davenport and Short [65]. Davenport and Short defined processes as “a set of logically-related tasks performed to achieve a defined business outcome”, and they stated that a company should redesign such processes when they prove to be inefficient or ineffective [65]. Their research proposed a methodology consisting on five steps: (i) Develop the business vision and process objectives; (ii) Identify the processes to be redesigned; (iii) Understand and measure the performance of existing processes; (iv) Identify IT levers; and (v) Design and build a process prototype and implement improvements (Davenport and Short, 1990). Harrington defined business process innovation as “a systematic methodology developed to help significant advances in the way its business processes operate”. His model is composed of five stages: (i) Organising for quality; (ii) Understand the process; (iii) Rationalise processes; (iv) Implement, measure, and monitor, and (v) Continuous Improvement [64]. The literature also presents different approaches for business process improvement (Hammer and Champy [66]; Elzinga et al., [67]; Lee and Chuah [68]; Gardner [69]; Alange and Steiber [70]; Page [71]; and [72], among others).

The next section describes the methodology of this research paper, including a justification for the selected business improvement process framework.

## 3. Methodology

The objective of this research is to develop and apply a process of innovation methodology based on Lean principles in the healthcare industry, in particular in hip fracture processes. In order to pursue the objective, a research design based on a case study and action research (AR) was developed.

A qualitative case study methodology is appropriate when there is an interest in knowing the “how” and “why” of a phenomenon and it is focused in contemporary events [73]. Theory built from cases is likely “to have important strengths like novelty, testability, and empirical validity, which arise from the intimate linkage with empirical evidence” [74], and it is also likely to be interesting, accurate and testable, as they use a wide range of data sources such as interviews, documentation, quantitative data, and direct observations [75].

Greenwood and Levin defined AR as “the research in which the validity and value of the research results are tested through collaborative insider-professional researcher knowledge generation and application processes in projects of social change that aim to increase fairness, wellness, and self-determination”. AR allows collaboration between professional researchers and community and organizational stakeholders in “defining the objectives, constructing the research questions, learning research skills, pooling knowledge and efforts, conducting the research, interpreting the results, and applying what is learned to produce positive social change” [76]. An AR methodology aims at simultaneously generating an action and building knowledge related to this action; thus, the results are both the action or intervention and the research itself [77,78]. Empirical AR is carried out, as the researchers document a current phenomenon, follow the process and share the results. It is also a participatory research, as the researchers maintain active and close contact with agents within the organization, as both are part of the research team [77]. AR is research in action, participative, concurrent with action and consisting of a sequence of events with a focus on problem solving [78].

Alfaro and Avella in 2013 proposed to conduct a preliminary stage in AR which consists of the identification of the problem or opportunity that the research team pretends to study; this should be done as a teamwork activity among researchers and practitioners [77]. Once the study topic has been identified, the six main steps in AR are data gathering, data feedback, data analysis, action planning, implementation, and evaluation [78].

A private general medical surgical hospital has been chosen as a case study. It is located in a +120,000 habitants city in the North of Spain, in a sparsely populated region. This study focuses on optimization and improvement in the design of hip fracture surgery processes in the above-mentioned hospital. The justification for the case study lies in the fact that the hospital is located in a region with a clear aging population and consequently there is a high prevalence of hip fracture cases. The hospital decided to start offering the service “hip fracture surgery” in 2019; thus, operating rooms were assigned, and professionals were hired for this purpose. Before 2019, this service was only provided in particular and punctual cases; since 2019 the demand forecast is 150 surgeries per year.

The case study hospital has a long trajectory of working according to high quality standards (as it is certified ISO 9001 and EFQM 500+). Moreover, it shows a strong commitment towards continuous improvement, as the new service “hip fracture surgery” implies high values of LOS of patients, as well as high resource consumption. This justifies the interest, both for the hospital and for the Public Health system, in studying, analysing, and proposing improvements in the process with the aim of improving the quality of the services provided, the efficiency of the operations, and the experience of the patients and their quality of life once they have left the hospital facilities.

In this research, AR is ensured by the collaboration of researchers (expert in Lean, process innovation, and healthcare) with hospital management and professionals (doctors, nurses, technicians), who jointly defined the research objectives, conducted the research, analysed and discussed the results, and planned for action implementation and the next steps.

The research team decided to use the framework based on process innovation by Suárez-Barraza et al. (2019) [72]. The main justification for this is that the selected model is based on Kaizen and Lean management systems [8,9] with a gradual and continuous improvement focus. On the other hand, other models are closer to engineering and are more appropriate for manufacturing operations, and are more oriented to breakthrough or radical innovation. Lean management systems originated in Toyota’s automotive factories, and they are used today in countless companies and organizations, both in manufacturing and services, having begun to be used in the healthcare sector in recent years [16,19,20]. Lean management systems seek to analyse production processes with the aim of identifying the activities that add value to the customer, and then minimizing or eliminating all the activities that do not add any value, called waste (*muda*, in Japanese).

**Figure 1:**
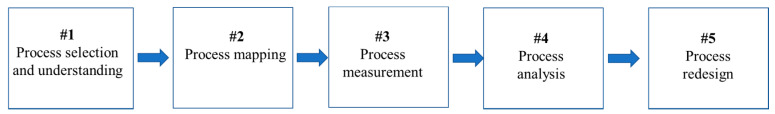
Process innovation framework.

The process innovation framework consists of the following stages [72], as shown in Figure 1: (1) process selection and understanding the process, (2) mapping the process, (3) process measurement, (4) process analysis, (5) process redesign [72].

Data analysis and collection

We selected the 2019 and 2020 years for observation because we wanted to know the details of the complete process related to hip fracture. Data and statistics were collected during 2019 and 2020. The field work and analytical phase occurred from November 2019 to March 2020. We combined the statistical analysis of the hip fracture patient’s dataset attended over 2019 and 2020 with process innovation and Lean healthcare analysis, including user experience ones. In this case, the study combined administrative data, experts’ point of view, and an observational process review.

## 4. Case Study: Hip Fracture Surgery Description

In this section we present a general description of the hospital case study, as well as a summary of descriptive statistics about the hip fracture surgery process in 2019 and 2020.

The special features that make the case study relevant are related to the aging of the population, the depopulation of this region, social isolation, and the lack of rural doctors and health care centers. The importance of the management of patient´s admission after hip fracture and discharge is undeniable. The case study hospital counts with over 300 beds, and provides medical services in close to 40 disciplines, which include traumatology, geriatrics, and cardiology, among others.

The hospital, immersed in a process of continuous quality improvement, analyzed the 2019 data and consequently took some actions oriented to improve them. Actions consisted of standardizing the medical-surgical process, consolidating an orthogeriatrics team, and carrying out an exhaustive follow-up. According to the data described below, a high impact was achieved, resulting in a reduction in average stays and mortality. The research described in this paper comes as the next step in this process of continuous improvement at the case study hospital, and consists of the analysis with Lean methodology with the aim of detecting potential points of improvement, within the real possibilities of the process at hand.

Hip fracture surgery at the hospital provides a service to 150 hip interventions annually as mentioned above in the case study justification in the Methodology section (Section 3).

A descriptive statistical analysis has been carried out to describe the population:

**Total patient population**. We reviewed 148 clinical record histories in 2019, from 39 men and 109 women. In 2020, we reviewed 106 clinical records, from 26 men and 80 women.

**Age**. Related to age, in 2019, the average age was 86.5 years, the minimum being 68.5 years and the maximum being 99.9 years. In 2020, the average was 85.5 years, the minimum being 66 years and the maximum being 101 years. By gender, in 2019 women/men average ages were 87/85 years, and in 2020 women/men average ages were 87/81 years.

**In-hospital mortality**. In 2019, in-hospital mortality was about 6.77% of admissions or 8.78% of patients (13 patients). Discharge due to death occurs at different times, on average two weeks after admission, although half of the deaths occur before 9 days. Moreover, 30 days after discharge, the mortality rate is 1.35% (2 patients). In 2020, in-hospital mortality was reduced to 3.7%.

**Total hospitalization time.** Total hospitalization time refers to the total process cycle time, from patient arrival to the hospital to the moment when the patient is discharged and leaves the hospital. In 2019, half of patients (median value for cycle times) were discharged within 11.9 days, the average being 15.9 days; 25% of patients were discharged at 16 days or more. In 2020, the LOS median value was 9 days, the average being 9.41 days (40.97% less than 2019 data).

**Presurgical time.** The average presurgical time (from patient admission to surgery) was approximately 4 days in 2019, half of patients underwent surgery in 3.16 days or less. During 2020, presurgical time was 3.15 days.

**Post-surgical average stay.** The average length of stay after surgery in 2019 was 10.12 days. A length of stay after surgery of more than 20 days could be considered exceptional. On the other hand, during 2020, the average post-surgical time was reduced to 5.25 days, a reduction of approximately 50%.

A summary of the descriptive statistics is shown in Table 1 below:

## 5. Applying Lean in Process Innovation in Hip Fracture: Results

### 5.1. Process Selection and Understanding the Process

**Figure A1:**
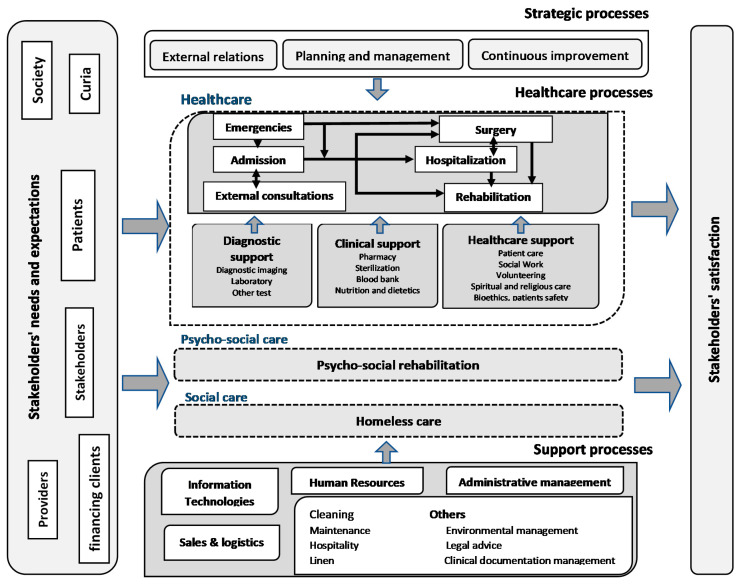
Source: Hospital Quality Management System.

The quality management system at the case study hospital has been built based on the analysis and understanding of the different needs and expectations of all the involved stakeholders (patients, society, suppliers, collaborators, finance clients) with the aim of providing them with the highest level of satisfaction. The system classifies internal processes in strategic processes, care processes, and support processes. Appendix A Figure A1 presents a detailed map of all of them.

Strategic processes refer to external relations of the hospital, to the management and planning, and to the continuous improvement.

Care processes refer to health attention, psychological attention, and social attention. Health attention consists of all the processes oriented to provide a health service to the patients, involving health operational steps (such as emergency, admissions, external medical consultations, hospitalization, surgery, rehabilitation), but also to diagnosis support (laboratory, image, other tests), clinical support (pharmacy, sterilization, blood reserves, nutrition, and dietetics) and care support (patient care, social work, volunteering, spiritual and religious care, bioethics, and patient safety). Psychological attention consists of all the processes oriented to provide a psychological rehabilitation service. Social attention consists of all the processes oriented to provide a service to homeless patients.

Support processes refer to information technologies, procurement and logistics, human resources, administration, and other processes.

### 5.2. Mapping the Process

The research team conducting this project is formed by the paper authors, two of them being experts in healthcare processes (in particular, in hip fracture) and two of them being experts in Lean management and innovation process methodologies. Collaboration with hospital management and hospital professionals (technicians, nurses, doctors) has been necessary in this research; in particular, the collaboration of a traumatologist doctor who is in charge of hip fracture surgery. Mapping the process refers to the documentation of the present situation and to the identification of flow.

Documenting the present situation must be done as it is in reality and not under ideal conditions. This has been conducted in two steps: first, the experts in hip fracture wrote the flow or sequence of activities from the first to the last step, i.e., from the first moment a patient enters the hospital until the moment the same patient leaves the facilities of the mentioned hospital. Second, a mixed team (expert in hip fracture and expert in Lean implementation) visited the hospital and followed the process from the first to the last activity, taking notes and pictures, asking questions to the different participants in the process activities, and walking the distances all along the path.

**Figure 2:**
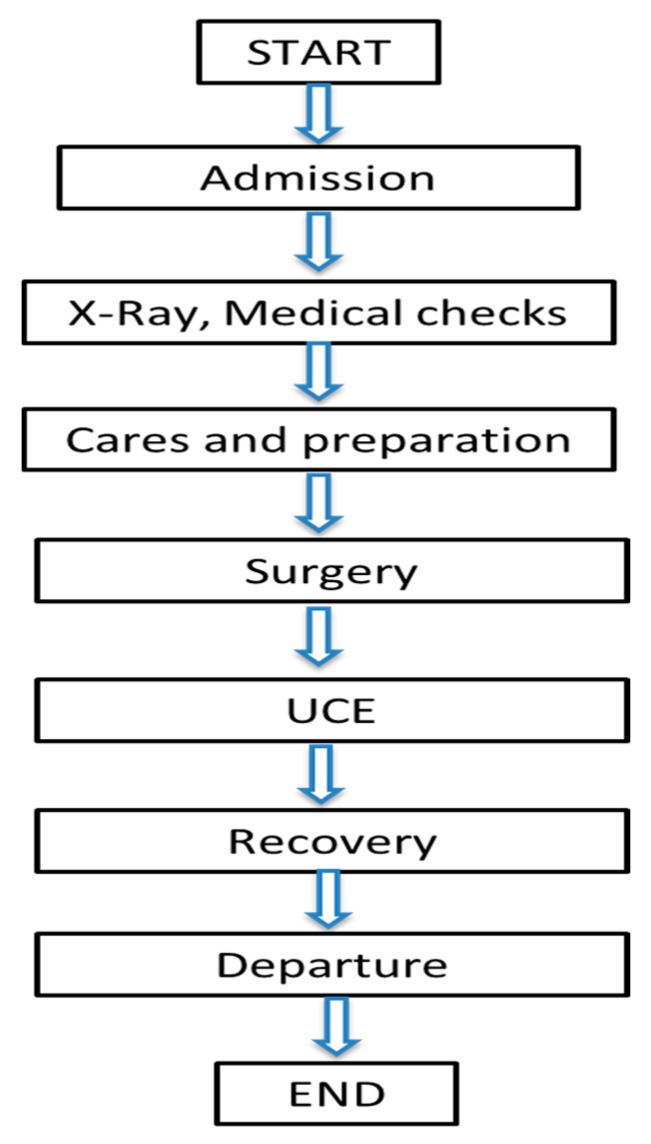
Block diagram.

**Figure 3:**
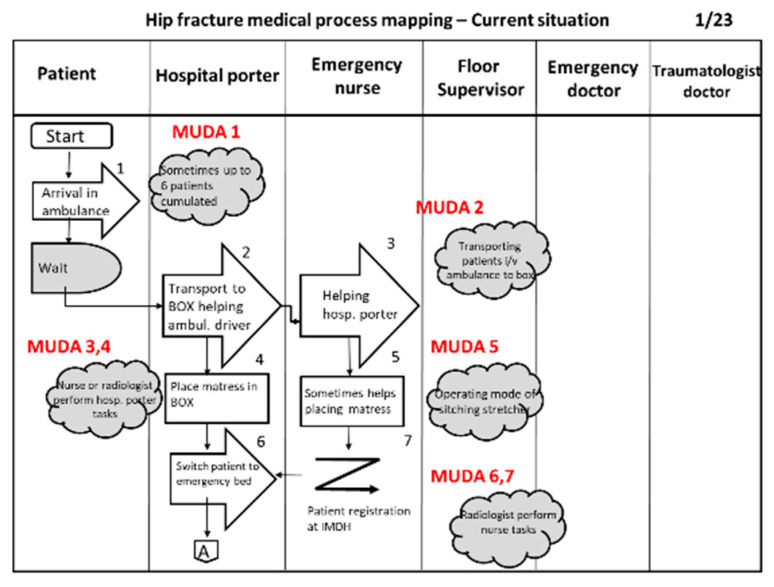
Flow diagram (partial) showing the existing process.

**Figure A2:**
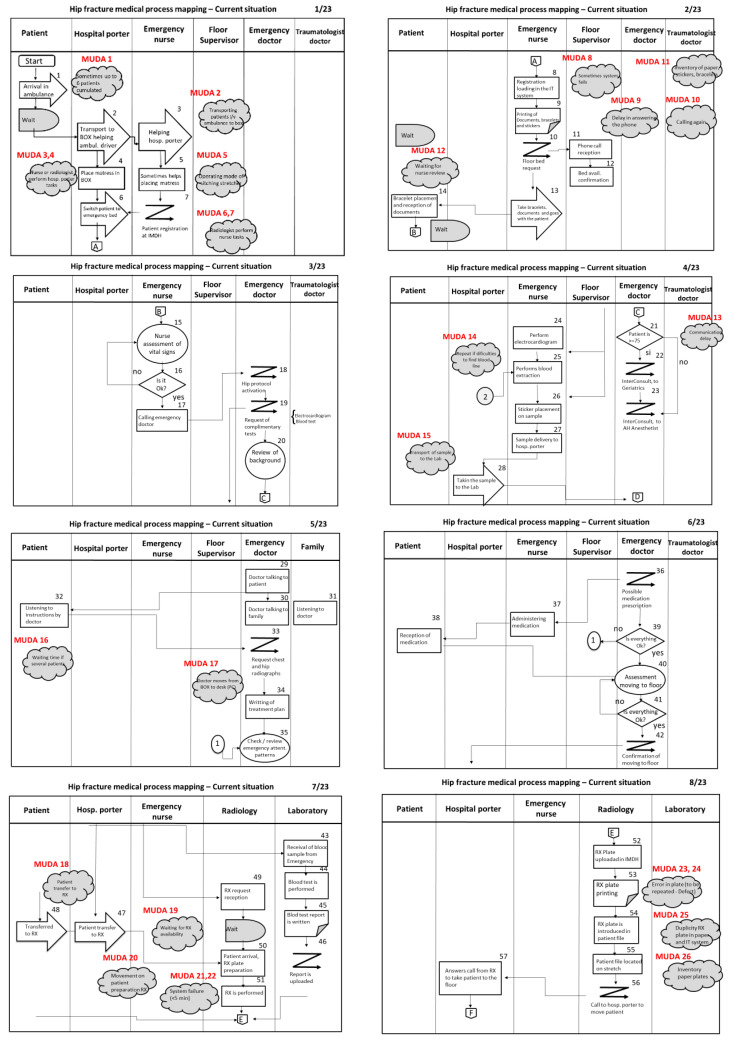

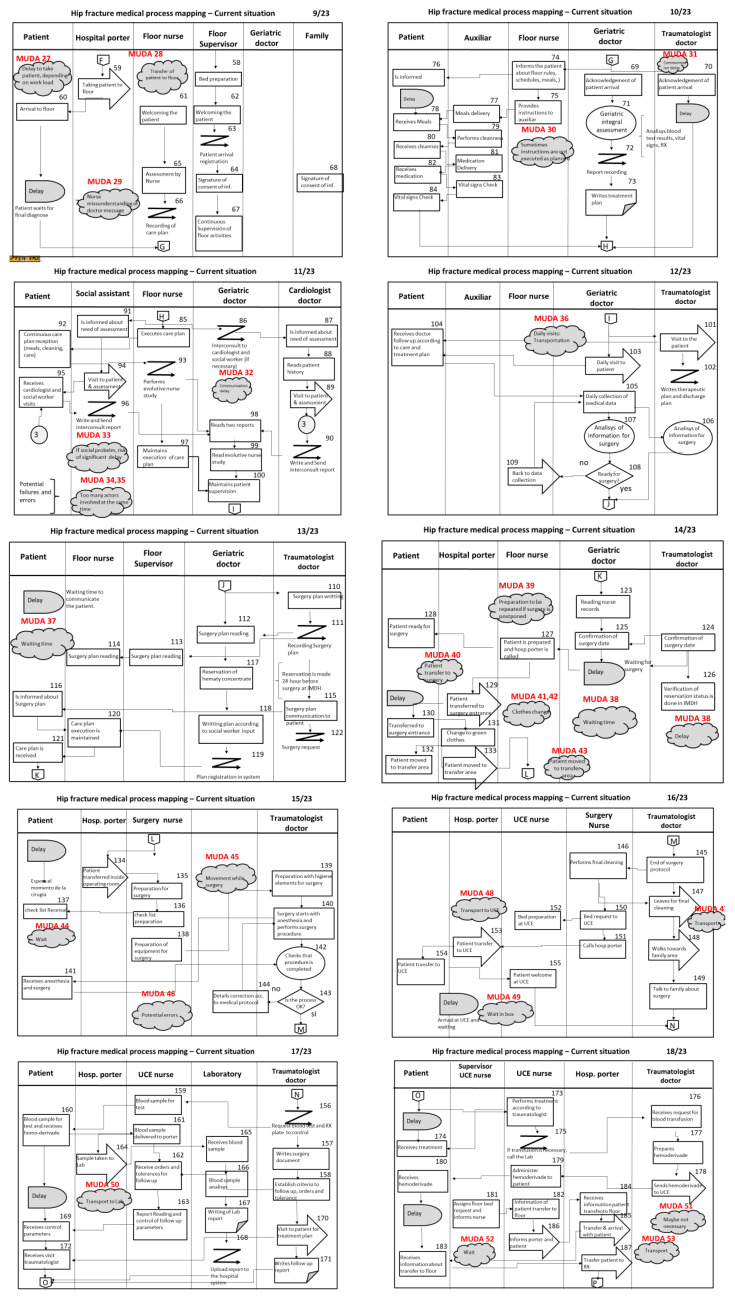

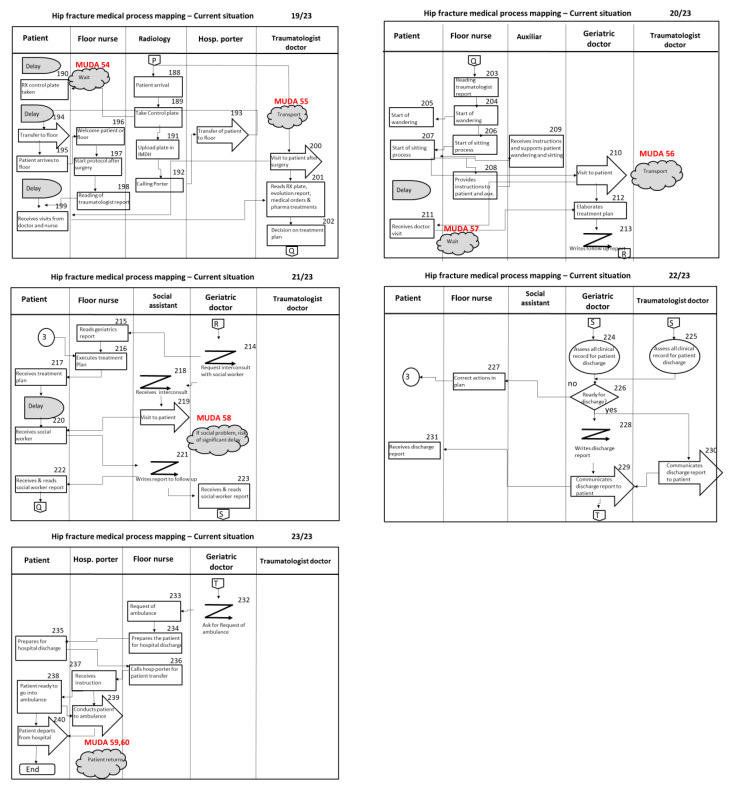
Hip Fracture Process Flowcharts.

The results of these two steps are the design of a block diagram (which provides a general description of the sequence of the process) and, based on it, the detailed process mapping using flow diagrams. Figure 2 shows a block diagram, which is the first step in the process analysis. The flow diagram of the hip fracture process uses symbols according to the American National Standard Institute (ANSI). Figure 3 presents an excerpt of a flow diagram of the process. This first excerpt contains only 7 activities of a total of 236. The complete flow diagram can be found in Appendix B Figure A2. The flow diagram was built on 23 pages that can be seen in Appendix B, showing that the hip care process map is a complex process in its current situation.

### 5.3. Process Measurements

Measuring the existing process implies identifying different indicators related to the process with the aim of quantifying them. Process redesign will propose some initiatives oriented to improve the values of the proposed metrics. The indicators that we consider are:

(i) Number of activities in the process. The number of activities is a relevant metric, as it is a measure of the complexity of the process. Each activity in the process has been identified and numbered.
  The total number of activities resulted in 236.
(ii) Number of participants. The number of participants is also a relevant metric, as a high number shows a more complicated process, as the number of interactions between them is higher, so there is a higher opportunity or risk of miscommunication, misunderstanding, delays, and potential errors. We understand the “number of participants” as the number of different jobs or profiles of employees, but the real number of employees intervening in the process is much higher. For instance, “nurse” is considered one participant, but in reality, we can find different persons working as nurses involved in the different activities of the process (same for doctors, assistants, technicians, etc.)
  The number of participants is 18 (*).
  They are grouped in different areas (see detail in Table 2).
(iii) Process cycle times. This refers to the total process time, from patient arrival at the hospital to the moment that the patient is discharged and leaves the hospital (also known as total hospitalization times or LOS). Total process cycle times in 2020 are reported above in the case study description, the average being 9.41 days (40.97% less than 2019 data), the median being 9 days, the standard deviation being 4.17, the minimum being 0 days, and the maximum value being 32 days.

### 5.4. Process Analysis

The process analysis is carried out in two steps:

(i) First, the process is analyzed in detail, identifying the different types of activities within the process. The following Table 3 provides this information:
(ii) Second, the process is analyzed with the aim of identifying the different types of *muda* all along the different stages. According to the literature, the following types of *muda* can be found in a process:
  - Defects: errors or defects when performing a task, producing a service or making a product.
  - Overprocessing: repeating tasks or activities during the process.
  - Overproduction: producing more than necessary.
  - Movement: unnecessary or inadequate movement of personnel to execute a task (related to ergonomics or efficiency of movements).
  - Transportation: carrying out or moving materials or patients from one location to a different location where a new task will be performed. This also refers to the transportation of employees.
  - Inventory: excess of materials or goods which are cumulated in case they are needed.
  - Delay: additional waiting time when the process stops more than usual.
  - Unused Talent: underused qualified workers (it could also be—but not in this case—a bad attitude from employees affecting the process results).

Our analysis shows that 60 opportunities for potential *muda* have been identified in 54 activities (i.e., 6 activities present 2 types of *muda*). Table 4 shows a summary of the different types of *Muda*. Appendix C Table A1 provides the details of all of them.

### 5.5. Process Redesign

AR is involved in the teamwork activities carried out between researchers and practitioners. Process measurements and analysis have been conducted by the research team (based on its experience in Lean, process innovation, and healthcare) with the collaboration of the hospital director, hospital employees (nurses, technicians), and mainly by the active support and involvement of a traumatologist doctor who is also in charge of hip fracture surgery at the hospital.

After process analysis has been conducted by the research team in collaboration with hospital professionals (management, doctors, nurses), process redesign will consist of the proposal and implementation of some initiatives oriented to enhance value creation from the patient point of view, i.e., reducing waste, increasing efficiency, improving patient experience, and improving the values of the process metrics. Table 5 below summarizes a list of actions that have been proposed for further implementation, identifying the type of *muda* that could be impacted by them:

As a next step, beyond the scope of this research paper, the authors suggest that an implementation plan could be designed, scheduled, executed, and evaluated. A new multidisciplinary team (involving researchers and hospital professionals) could be formed with the aim of guaranteeing that expected results are confirmed.

## 6. Discussion

The results of the care activity of the case study hospital are similar in average age to those found in the national hip fracture registry in Spain, with an average age of 87 years [35]. The average LOS of this registry is 10 days, slightly higher than the LOS of the analysed hospital.

The research questions that govern this study are (i) to try to identify the types of waste or *muda*, and (ii) to evaluate the application of a process innovation approach in healthcare, in particular in the hip fracture surgery process.

This research allowed us to identify 60 *muda* opportunities along the hip fracture surgery process, which is composed of 236 activities and where 18 participants interact. It is relevant to point out that 18 participants does not refer to 18 people, but to 18 job positions, each of them being performed by 1 or more people. For instance, one position is a nurse; if the patient stays at the hospital for 2 weeks, there will be different people working as nurses to take care of him (the nursing team is composed of more than 10 employees per shift, morning, evening, and night, 24 h a day).

The following different types of *muda* have been identified along the process: defects, overprocessing, overproduction, movement, transportation, inventory, delay, and unused talent. Some of them occur punctually, but others (we consider them as critical *muda*) are more repetitive all along the process; these are the ones that we are going to discuss. Although three types of *muda* represent 72% of the total (enough for critical *muda* discussion), we also decided to consider the fourth type (which only represents 10% of the total).

Delay is the most frequent waste in the hip fracture surgery process. Delay represents 33% of all cases, the most common being as follows: (i) the patient needs to wait because the clinical process requires it (for instance, the patient needs to be stabilized—pulse, temperature, blood pressure, etc.—before being transferred to the next stage); (ii) the patient needs to wait because of a lack of available resources (no available hospital porter for transfer) or because all resources are busy (for example, X-Ray is being provided to a different patient; (iii) delays produced because an employee has forgotten or neglected a task, or presents a bad attitude. Type (i) is normal; type (ii) needs to be minimized (better resource planning); type (iii) needs to be eradicated. Communication between employees patients is crucial in each case because, even if the delay cannot be avoided, the patient experience could be positive if there is a clear explanation and justification for it.

Transportation is the second type of waste. In general, transportation refers to transferring the patient from one location to a different location, and, although frequent, it is not very relevant, as distances within the hospital are not big. When referring to transportation of employees (doctors, nurses, porters, etc.) there is evidence of inefficiency (repeated distances are walked to supervise, take care, provide service etc., to patients). Spaghetti charts and cause-and-effect analyses could be used to study this type of *muda* in detail.

Overprocessing waste could happen when (i) some tasks need to be repeated; (ii) the process is not well- or properly defined; or (iii) lack of resources or when employees perform tasks that are not supposed to be carried out by them. Waste reduction could be achieved by different means such as automation (e.g., when initiating the hip fracture process the system automatically requests a bed, avoiding the need to phone or call a floor supervisor), process standardization with poka-yokes or checklists (e.g., to avoid repeating blood tests or X-Rays), better planning (e.g., to avoid repeating the pre-surgery preparation); or better maintenance planning (e.g., reliable maintenance plans avoid equipment and machinery failures).

Defect refers to waste when the results of an activity are not right, i.e., when an error is produced. Errors could be clinical, or cause by service, attention, etc. Causes of errors could be diverse, but if clinical errors are excluded, many are related to miscommunication (too many participants in the process), a lack of a standardized processes (every employee adopts his/her own criteria to execute an activity or to take a decision), or to an excessive workload (attention and concentration decrease). A leaner process with less participants, standardized processes (process defines, trained employees, checklists, and poka-yokes) and a planned workload will reduce waste opportunities.

An inventory of materials is necessary at many stages in the process: labels, paper and bracelets in admissions; and X-Ray plates, medicines, and medical goods in emergency areas, rooms, UCEs, and operating rooms. Every supervisor is responsible for inventory management (inventory levels, keeping control, placing the orders). No specific method (economic order quantity, fixed period ordering, first in first out, etc.), nor software is used, but a manual control is carried out according to his/her own criteria. Lack or excess of an inventory has been reported, an excess of it being more frequent due to the fact that healthcare deals with patient lives and risks should be minimized. There is a pharmacy inside the hospital, in case of any material need.

Movement waste in this process deals mainly with patient manipulation to be transferred from a stretcher to accommodate a mattress, the handling of X-Rays, electrode tests, blood test sampling, or movement inside the operating room. Process standardization according to different patient types (weight, volume, health condition) would help to minimize this *muda*.

Different types of *muda* according to the literature [8,9,11,12] have been identified in the process, being delay the most frequent. This agrees with Godley et al. [60] and Woodnutt [7], who identified delay and waiting times as the relevant types of waste that should be addressed with the aim of optimizing processes. The literature shows different examples of authors who have studied the different types of *muda* in the same way. In the educational sector, Doman indicates that with process innovation efforts, the graduate and graduate management processes can be improved [79]. Walters et al. identified specific areas of internal production waste including defects and waiting, and in the process of our investigation, identified a significant shift in process efficiency due to resource allocation [80] focusing on 1040 financial norms. In fact, Ann Douglas et al. similarly identified the 8 types of *muda* in Higher Education [81] and Suárez-Barraza et al. also identified three new types of *muda* in the 21st century such as unnecessary emails, excess work meetings, and technological distractors [82]; processes in hospitals cannot be free of these three new types of *muda* either.

Finally, Coelho et al. present evidence of process innovation in health processes, for example, they reduced the cancer outpatient chemotherapy process cycle time from 2 h to 30 min [57].

Close collaboration between clinical staff, hospital management, and researchers allowed the collection of precise data, as well as information sharing, which is very valuable for the process analysis and redesign. A new process design presents the following advantages, which are aligned with the literature [31,42,44,60]:

For patients, a reduction of LOS, potential errors, and, as a consequence, an improvement in patient satisfaction and experience. Quality improvement impacts directly on the quality of life of the patient, including both psychological and social aspects related to the social isolation in which the patient returns after discharge.

For hospital management, an increase in efficiency and better planning, thus a cost reduction, a capacity increase, and, as a consequence, a potential increase of activity.

For hospital employees, waste reduction (times, overprocessing, potential errors, etc.) and standardized processes will reduce the workload, stress and fatigue, increasing their satisfaction, thus, their motivation and commitment.

The above-mentioned findings justify the selection of this process innovation framework, as it has a clear focus on waste identification and Lean operations redesign [72]. The identification of changes in the analyzed process with the proposed methodology involves a second phase of analysis and reflection with the orthogeriatric team to go in depth into the findings, and to identify those that can really undergo an improvement process.

## 7. Conclusions

The research examines the identification of waste or *muda* in a hip fracture surgery process in healthcare. Eight types of *muda* have been identified, the most frequent being delay, transportation, overprocessing, and defects, and actions based on them for improvement have been proposed.

The application of a process innovation approach has also been examined in this research, the result being that, although innovative, this approach is appropriate for the healthcare sector, as it is appropriate in any other service industry. Applying the process innovation methodology represents an effort of the Kaizen philosophy in critical processes in the health sector. In fact, it allows crystallizing redesigns and changes to eliminate *muda* from the activities of health processes. In our case study, all the proposals will lower the *muda* percentage by at least 30% for all hip process activities in the current situation.

This research is innovative on the implementation of the technical approach, and its contributions on the implementation side can be summarized by a set of proposals that have been done including: process standardization, reduction of the number of participants in the process, techniques to improve communication, automation initiatives, training, implementation of inventory management techniques, the implementation of some tools (such as 5 s, checklists, or poka-yokes), including new performance indicators, as well as patient satisfaction measurement systems, among others.

This research is also innovative on the action-research process itself, as it brings the following contributions: (1) although not common, we succeeded in obtaining a formal collaboration among clinical personnel (doctors, nurses, etc.), service managers, and researchers to carry out a joint research project; (2) a research team (expert in Lean and process innovation, as well as in healthcare) entering the hospital and visiting the whole detailed process overcoming potential problems or communication barriers; (3) the advantages of documenting and recording observations and results (the power of direct observation in the *gemba*, the Japanese word for “place”); (4) fruitful discussions among specialists, experts, managers, and researchers towards sharing knowledge and ideas aiming at process improvement; (5) the adaptation of a process innovation approach within the healthcare sector, which is different from other service industries due to different needs, priorities, and vocabulary, as well as differences between private or public hospitals.

When the average stay data is adjusted to normal [35], it can be complex to introduce improvements that contribute to the sustainability of the service and increase the impact on the patient. Therefore, an exhaustive analysis of each one of the *mudas* found before their implantation is necessary.

This research also offers some practical implications for healthcare managers:

(i) the identification of all different types of *muda* all along the hip fracture surgery process provides hospital managers with an opportunity for continuous improvement, by trying to eliminate or minimize them;
(ii) some initiatives have been proposed to redesign the process, which allows the management to take action towards gaining efficiency and service quality, which in turn impacts on operating costs and patient satisfaction;
(iii) the observation and the analysis have been carried out by researchers, but a basic training and a checklist (an audit tool) would help employees to carry out this assessment any time they need in the future.

Finally, some limitations of the research are: (i) the data cannot be generalized due to its qualitative nature; (ii) the findings refer to the specific context of hip fracture surgery in a case study in Spain; (iii) process redesign initiatives have been proposed for implementation, but have not been evaluated yet. Future studies could be carried out with the objective of evaluating the effectiveness of the implemented actions and their outcome, as well as by using data analysis techniques to better understand the variability of data dispersion. Other future areas for research could be initiated using several years of data history in the case study, and by using a larger sample of hospitals, as well as other geographical areas.

## Figures and Tables

**Table 1:** Summary of descriptive statistical analysis.

	2019		2020	
	Average	Std Dev	Average	Std Dev
Total patient population				
Total (Women/Men)	148 (109/39)		106 (80/26)	
Age (years)	86.55	6.34	85.48	6.77
In-hospital mortality (%)	6.77		3.7	
Total hospitalization time (days)	15.9	15.36	9.41	4.17
Presurgical time (days)	3.98	3.24	3.15	2.55
Post-surgical average stay (days)	10.12	6.77	5.25	3.25

**Table 2:** Detail of areas and participants in hip fracture surgery processes.

General:	Emergency Area:	Surgery:
Patient Family Traumatologist doctor (*) Cardiologist doctor Laboratory technician X-Ray technician Social assistant Hospital porter	Emergency nurse Emergency doctor	Surgery nurse Surgery hospital porter
	**Hospital floor level:**	**UCE (short stay unit):**
	Floor nurse Floor nurse supervisor Floor assistant Geriatric doctor	UCE nurse UCE supervisor

Note (*): It is important to acknowledge that the traumatologist doctor also acts as the surgeon.

**Table 3:** Analysis of hip fracture process activities.

Activity Type	Flowchart Symbol	Count
Activity	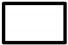	151
Delay	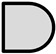	20
Transport	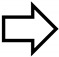	35
Decisions	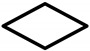	7
Inspection	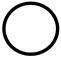	10
Internal document	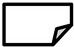	6
Electronic transport	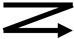	31
Type of Muda	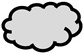	60
Total	Process activities Muda	240 60

**Table 4:** Summary of the different types of Muda: (**a**) Data, (**b**) Pareto chart.

**Type of Muda**	**Count**	**%**	**Cumul %**	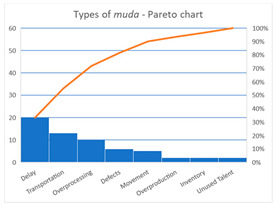
Delay	20	33%	33%	
Transportation	13	22%	55%	
Overprocessing	10	17%	72%	
Defects	6	10%	82%	
Movement	5	8%	90%	
Overproduction	2	3%	93%	
Inventory	2	3%	97%	
Unused Talent	2	3%	100%	
Total	60	100%	100%	
(**a**)				(**b**)

**Table 5:** List of proposals.

Proposals	*Muda*
Establish a communication protocol with the Public Health system to ensure that every new patient brings a medical history file (in paper or electronic format), thus a more efficient approach will be adopted (some tests might not be necessary, avoiding duplicity and improving patient experience).	Delay Transportation Overprocessing Movement Overproduction
Reduce the number of activities and the number of participants. Ex. 1. Portable X-Ray equipment would help patients to be transferred from emergency area directly to floor, as X-Ray test could be carried out at that moment. Ex. 2. Transfer of patient to operating area to be done in a way that only one hospital porter is required to dress/undress with safety protection equipment.	All 8 types of *muda*
Avoid duplication of tasks (for instance, printing X-Ray in paper and saving it electronically).	Delay Overproduction
Reduce distances by locating some areas in a different place. Ex. After surgery doctor speaking to patients in a meeting room.	Delay Transportation
Review and maintenance of electronic equipment and IT systems with the aim of detecting potential causes for malfunctioning.	Delay Overprocessing
Automate some steps: When starting the process, some actions could be automatically initiated as tests (blood test, X-Ray) and medical consultations (calls to geriatrics or cardiologist).	Delay Overprocessing
Implement a system to measure the quality of the delivered service (patient satisfaction, patient experience, etc.).	Overprocessing Defects
Implement poka-yokes in operating processes within the operating room: marking leg, surgery checklist to be displayed in operating area, screen or board with main steps identifying critical activities with symbols.	Overprocessing Defects Overproduction
Implementation of 5s techniques in operating processes within the operating room.	All 8 types of *muda*
Clinical process standardization (for surgery, doctors and nurses). Administrative and data input process standardization. Training of personnel according to the abovementioned new standardized processes.	All 8 types of *muda*
Implementation of communication practices between doctors and nurses based on documentation of orders and instructions (it could be in the computer system or in patient´s logbooks) with the aim of minimizing errors due to misunderstandings or wrong interpretations.	Transportation Overprocessing Defects Overproduction
Implementation of an adequate inventory management system (EOQ, FIFO, etc.).	Inventory
Implementation of tools to record and share best practices within the hospital and among different hospitals within the group. Further data analysis to understand the variability of data.	All 8 types of *muda*

## Appendix A

Main Process s at case study hospital.

## Appendix C

**Table A1:** Analysis of different types of *muda*.

Muda #	Process Activity #	Direct Observation	Type of Muda
1	1	Ambulance arrival. Patient cumulation (sometimes up to 6 patients)	Delay
2	3	Transportation of patients from ambulance to emergency box	Transportation
3, 4	4	MRI technician or nurse working as a hospital porter if required	Unused Talent Overprocessing
5	5	Handling patients from an ambulance stretcher to a hospital stretcher and placing mattress	Movement
6, 7	7	MRI technician working as a nurse if required to register patients	Unused Talent Overprocessing
8	8	Registration activities to be redone when IMDH systems fail	Overprocessing
9	11	Waiting time while phone is answered	Delay
10	12	Phone calls to be repeated until somebody answers	Overprocessing
11	9	Excessive or potential lack of inventory of paper, labels, or bracelets	Inventory
12	14	Waiting for a nurse check	Delay
13	21	Communication delay	Delay
14	25	Blood extraction preparation to be repeated in the case of difficulties	Overprocessing
15	28	Transportation of blood sample	Transportation
16	32	Waiting time if several patients are in the emergency area	Delay
17	34	Unnecessary doctor movement between box (patient) and desk (PC)	Movement
18	48	Transportation of patients from emergency box to X-Ray	Transportation
19	47	Waiting times upon X-Ray availability	Delay
20	50	Handling of patients for X-Ray preparation	Movement
21, 22	51	System error provoking delay (up to 5 min) and repeating operations	Delay, Overprocessing
23	53	Defective X-Ray	Defect
24	53	Repeated defective X-Ray	Overprocessing
25	54	Duplicity X-Ray in computer and in paper	Overproduction
26	55	Inventory of paper X-Ray plates	Inventory
27	59	Waiting times to take patient to room	Delay
28	60	Transportation of patients from X-Ray to room	Transportation
29	65	Error in understanding doctor’s orders	Defect
30	79	Deviations from original message given to patients	Defect
31	70	Communication delay	Delay
32	86	Communication delay	Delay
33	96	If social issue, high risk of huge delays	Delay
34, 35	85–100	Many actors, potential failures and errors	Defect, Overprocessing
36	101	Daily visits to patients	Transportation
37	114	Waiting for a decision	Delay
38	126	Waiting for transportation to surgery area	Delay
39	127	If surgery is delayed or postponed, patient preparation must be redone	Overprocessing
40	129	Transportation of patients to surgery level	Transportation
41, 42	131	Clothes changing	Movement, Delay
43	133	Transportation of patient to surgery transfer area	Transportation
44	137	Waiting for surgery	Delay
45	135–143	Movement and preparation before and during surgery	Movement
46	143	Potential errors during surgery	Defects
47	147	Doctor transportation for cleaning and talking to the patient family	Transportation
48	153	Transportation of patient to UCE	Transportation
49	155	Waiting time if busy area	Delay
50	164	Transportation of blood sample to Laboratory	Transportation
51	178	Homoderivative might not be necessary	Overproduction
52	183	Delay	Delay
53	187	Transportation of patient to X-Ray	Transportation
54	190	Delay	Delay
55	193	Transportation of patient to room	Transportation
56	210	Daily visits to patients	Transportation
57	211	Delay	Delay
58	218–221	If social issue, high risk of huge delays	Delay
59, 60	240	Patient returns sooner than expected due to a recovery issue	Defect; Overprocessing
